# Retention of essential fatty acids in fish differs by species, habitat use and nutritional quality of prey

**DOI:** 10.1002/ece3.10158

**Published:** 2023-06-01

**Authors:** Tharindu Bandara, Sonia Brugel, Agneta Andersson, Danny Chun Pong Lau

**Affiliations:** ^1^ Department of Ecology and Environmental Science Umeå University Umeå Sweden; ^2^ Department of Animal Science, Faculty of Animal Science and Export Agriculture Uva Wellassa University Badulla Sri Lanka; ^3^ Umeå Marine Sciences Centre Umeå University Hörnefors Sweden; ^4^ Department of Aquatic Sciences and Assessment Swedish University of Agricultural Sciences Uppsala Sweden

**Keywords:** Baltic Sea, benthic macroinvertebrates, DHA, perch, polyunsaturated fatty acids, roach, trophic transfer, zooplankton

## Abstract

Algae‐produced long‐chain polyunsaturated fatty acids (LC‐PUFA; with ≥20 carbon atoms) are key biomolecules for consumer production and animal health. They are transferred to higher trophic levels and accumulated in food chains. However, LC‐PUFA accumulation in consumers and their trophic transfer vary with the diet quality and the physiological demand for LC‐PUFA of consumers. The goal of this study was to investigate spatial and taxonomic differences in LC‐PUFA retention of coastal fish predators that potentially differ in their habitat use (benthic versus pelagic) and prey quality. We analyzed the fatty acid (FA) composition of common fish species, namely roach and European perch, as well as their potential prey from benthic and pelagic habitats in three bays of the northern Baltic Sea. We then assessed whether the fish LC‐PUFA retention differed between species and among the study bays with different diet quality, that is, LC‐PUFA availability. Our data indicated taxon‐specific differences in the retention of LC‐PUFA and their precursor FA in fish (i.e., short‐chain PUFA with <20 carbon atoms). Perch did not show any spatial variation in the retention of all these FA, while roach showed spatial differences in the retention of docosahexaenoic acid (DHA) and their precursor FA, but not eicosapentaenoic acid (EPA). Data suggest that diet quality and trophic reliance on benthic prey underlay the DHA retention differences in roach. Although the PUFA supply might differ among sites, the low spatial variation in LC‐PUFA content of perch and roach indicates that both fishes were able to selectively retain dietary LC‐PUFA. Climate change together with other existing human‐caused environmental stressors are expected to alter the algal assemblages and lower their LC‐PUFA supply for aquatic food webs. Our findings imply that these stressors will pose heterogeneous impacts on different fish predators. We advocate further investigations on how environmental changes would affect the nutritional quality of the basal trophic level, and their subsequent impacts on LC‐PUFA retention, trophic ecology, and performance of individual fish species.

## Graphical abstract

We investigated the spatial and taxonomic differences in algae‐produced long‐chain polyunsaturated fatty acid retention (i.e., LC‐PUFA; with ≥20 carbon atoms) in common fish predators (i.e., European perch and roach) in the coastal areas of the northern Baltic Sea. Our data indicated taxon‐specific differences in the retention of LC‐PUFA and their precursor FA (i.e., short‐chain PUFA with <20 carbon atoms) in fish. Perch did not show any spatial variation in the retention of all these FA, while roach showed spatial differences in the retention of docosahexaenoic acid (DHA) and their precursor FA. Our data suggest that diet quality and trophic reliance on benthic prey underlay the DHA retention differences in roach. Climate change together with other existing human‐caused environmental stressors is expected to alter the algal assemblages and lower their LC‐PUFA supply for aquatic food webs. Our findings imply that these stressors will pose heterogeneous impacts on different fish predators.

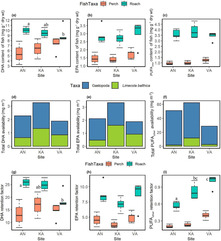

## 1 INTRODUCTION

Nutritional quality of diet is a major determinant of animal health and food web efficiency (Brett & Müller‐Navarra, 1997; Müller‐Navarra et al., 2000; Sargent et al., 1995). Among other biomolecules that indicate nutritional quality of the food resources, the polyunsaturated fatty acids (PUFA) in the omega 3 and omega 6 families are necessary for animal physiological functions. The long‐chain PUFA (LC‐PUFA) that have ≥20 carbon atoms, such as docosahexaenoic acid (DHA, 22:6 ω3) and eicosapentaenoic acid (EPA, 20:5ω3), are particularly important for regulating cell membrane fluidity, growth, reproduction, and immune response in animals (Brett & Müller‐Navarra, 1997; Fritz et al., 2017; Twining et al., 2016). These LC‐PUFA are mainly produced de novo by algae in aquatic environment (Napolitano, 1999). Algal taxa such as diatoms (Bacillariophyta) are rich in EPA, while cryptophytes and chrysophytes are considered DHA‐rich resources (Brett & Müller‐Navarra, 1997; Taipale et al., 2016). Algae‐produced DHA and EPA are transferred to the upper trophic levels via primary consumers (e.g., herbivorous zooplankton), and subsequently accumulated in aquatic food chains. Recent studies also suggest potential PUFA synthesis in several invertebrate taxa, as they contain the genes for enzymes that desaturate and elongate precursor FA into LC‐PUFA (Kabeya et al., 2018; Monroig et al., 2022). Nevertheless, aquatic consumers still heavily rely on the dietary supply of LC‐PUFA and/or their precursor fatty acids (FA) for LC‐PUFA synthesis (Twining et al., 2021).

Due to the physiological importance of DHA and EPA, they are selectively retained in fish (Arts & Kohler, 2009; Parrish, 2009). Generally, LC‐PUFA retention in fish can be influenced by their diet (Tocher, 2003), taxonomic identity, sex, reproductive stage, feeding guild, and the environmental conditions (Bell & Tocher, 2009; Guo et al., 2017; Scharnweber et al., 2021). Some fish species also have the ability to convert short‐chain PUFA obtained from diet into LC‐PUFA, but marine fish generally are less efficient in this bioconversion than are freshwater fish (Monroig et al., 2013; Twining et al., 2016). It is therefore expected that marine fish are more dependent on dietary LC‐PUFA inputs than are freshwater fish.

Roach (*Rutilus rutilus* [Linnaeus, 1758]; hereafter roach) and European perch (*Perca fluviatilis* Linnaeus, 1758; hereafter perch) are common fish predators in northern aquatic ecosystems including temperate lakes and the Baltic Sea coast, where climate change and other human‐caused environmental stressors are expected to reduce the LC‐PUFA production and transfer in the food webs (Bandara et al., 2022; Holm et al., 2022; Lau et al., 2021). Roach is a typical generalist and perch exhibits size‐dependent ontogenetic changes in feeding. Juvenile perch mainly depend on zooplankton, then shift to increasingly rely on benthic invertebrates and later on fish as they grow bigger (Estlander et al., 2010; Persson, 1988). However, adult perch individuals could still feed on a mixture of prey that satisfy their nutritional requirements (Scharnweber et al., 2016). It has been shown that diet contributes to most of the variation in the FA composition of perch (Chaguaceda et al., 2020). However, both roach and perch are able to convert precursor dietary FA into LC‐PUFA (Chaguaceda et al., 2020; Taipale et al., 2022). Yet, it is not clear whether the LC‐PUFA retention and the underlying contribution of diet and/or FA bioconversion ability differ among co‐existing fish taxa, especially when these taxa potentially rely on different prey resources (e.g., benthic versus pelagic).

The Baltic Sea is one of the largest brackish water ecosystems in the world. Over the decades, climate change‐induced environmental stressors such as increased terrestrial runoff and organic pollutants have been affecting the Baltic Sea ecosystem (Andersson et al., 2015). In the northern Baltic Sea, these stressors have adverse effects on the algal assemblages, thus reducing the algal LC‐PUFA production and the nutritional quality of the lower trophic levels (Bandara et al., 2022). These negative changes potentially propagate to affect the nutritional quality and well‐being of the predators, for example, fish, which generally have greater LC‐PUFA demands than do the lower trophic levels (Lau et al., 2012; Strandberg et al., 2015). Therefore, we selected four bays of the northern Baltic Sea as our study systems to assess the spatial and taxonomic differences in LC‐PUFA retention of roach and perch. We predicted that (i) both fishes preferentially retain LC‐PUFA (i.e., DHA and EPA) over the precursor FA (i.e., short‐chain PUFA with <20 carbon atoms); (ii) the LC‐PUFA requirement and retention of fish are taxon‐specific; and (iii) intraspecific variation in fish LC‐PUFA retention is related to their habitat use (i.e., benthic versus pelagic prey reliance) and the spatial differences in nutritional quality (i.e., LC‐PUFA availability) of their prey.

## 2 MATERIALS AND METHODS

### 2.1 Study sites and design

**FIGURE 1:**
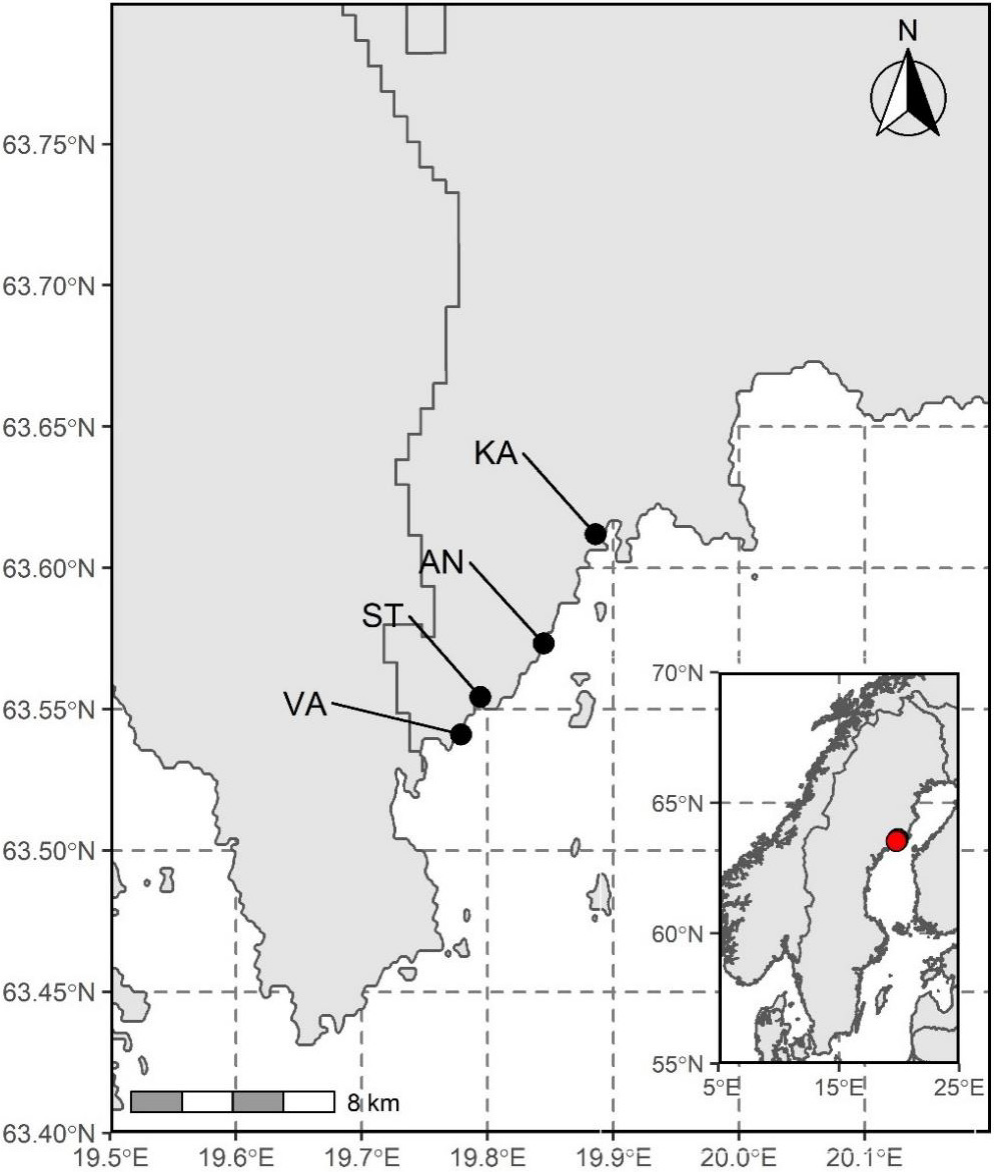
Locations of the study bays in the northern Baltic Sea. AN, Ängerån; KA, Kalvarsskatan; ST, Stadsviken; VA, Valviken.

We selected four bays in the northern Baltic Sea, namely Ängerån (63°34.400N, 19°50.666E), Kalvarsskatan (63°36.072N, 19°53.140E), Stadsviken (63°33.026N, 19°47.647E), and Valviken (63°32.468N, 19°46.725E), for this study (Figure 1). These bays are shallow and semi‐enclosed, with a maximum depth of 3–4 m at the offshore edge of the bay. They were relatively pristine and received limited impacts from human activities. The bays, however, received variable amounts of freshwater (river inflow and runoff) and dissolved organic carbon as well as nutrients from relatively undisturbed catchments. Freshwater input was largest in Ängerån and lowest in Kalvarsskatan (Guo et al., 2022), but summer 2018 was especially dry and warm in the area (weather records data at station Järnäsklubb A; SMHI, 2022), and the differences in freshwater input among the bays were then smaller than previous wetter years.

Our study included (i) water samples for analysis of the physicochemical characteristics of the bays, (ii) multiple taxa of benthic macroinvertebrates and zooplankton that were potential prey of fish, and (iii) two predatory fish species, namely roach and perch. Sampling at all bays was conducted between July and September 2018. Water and zooplankton samples were collected monthly, that is, three times in total. Benthic macroinvertebrates were collected once each in July and September, while fish were collected once in late August only. The collected animal samples were analyzed for stable isotopes and FA to determine the pelagic versus benthic prey reliance of fish and their FA retention (more detailed description below). The sampling frequency differed among animal groups because of resource and logistic constraints, and also because we considered that the rate of incorporating dietary isotopic signals in fish tissues (e.g., muscles) is much slower than that in their prey, due to the larger body mass of fish (Vander Zanden et al., 2015). Based on the equation provided by Vander Zanden et al. (2015) [ln(half‐life) = 0.22(ln(body mass)) + 3.28], the time required to reach 50% isotopic equilibration of our fish samples (muscles) with the diet was estimated to be 62 ± 11 days (mean ± SD; Vander Zanden et al., 2015). Our sampling design thus enabled us to detect potential temporal changes in isotopic and FA signals in only the fish prey, while fish were expected to have integrated these signals from their prey from at least July–August 2018. We then assessed FA retention in the fish in relation to the among‐bay differences in their habitat use (i.e., reliance on pelagic versus benthic prey) and the nutritional quality of their prey.

### 2.2 Physiochemical variables

We collected water samples from 0.5 m depth and filtered through 0.2 μm pore size Supor membrane filters (Acrodisc®, Pall). Total dissolved phosphorus (TDP) and total dissolved nitrogen (TDN) concentrations of the water samples were analyzed after oxidation with peroxodisulphate using a Seal QuAAtro39 auto‐analyzer (Grasshoff et al., 1983). Dissolved inorganic phosphorus (DIP) and dissolved inorganic nitrogen (DIN, i.e., nitrate, nitrite, and ammonium) concentrations of the samples were analyzed using a Seal QuAAtro39 auto‐analyzer (Grasshoff et al., 1983). After acidification with HCl (18 mM), dissolved organic carbon (DOC) concentration of the water samples was analyzed using a Shimadzu TOC‐5000 high‐temperature combustion analyzer. Water temperature, pH and salinity at 0.5 m below the water surface were measured using a conductivity meter (WTW ProfiLine Cond 3110, Germany).

### 2.3 Benthic macroinvertebrates, zooplankton and fish sampling

Benthic macroinvertebrates were collected using a Hydrobios Ekman bottom sampler at totally 3 sites (3 grabs per site) with a depth of 0.5, 1.5, and 2.5 m in each bay. The collected bottom samples were serially sieved on 10 and 0.5 mm, and the invertebrates were sorted by taxa and left in filtered seawater (0.2 μm) overnight at 4°C to empty their gut content. The invertebrates were stored at −20°C until freeze‐drying. The freeze‐dried samples were weighed to estimate the biomass of individual taxa, and then pulverized. We selected the common taxa for analysis of FA and stable isotopes. The selected taxa were dipteran Chironomidae, Gastropoda, bivalve *Limecola balthica* (Linnaeus, 1758), and polychaete *Marenzellaria* sp. We could not collect benthic invertebrates from Stadsviken because of the hard bottom substrates, and we did not calculate FA retention for the fish from this site.

Zooplankton samples were collected by horizontal net hauls in each bay, with a Hydrobios Apstein plankton net of 200 μm mesh size. The collected samples were left in filtered seawater (0.2 μm) overnight at 4°C to empty their gut content. Individuals were sorted by species and then freeze‐dried. The species present included *Eurytemora affinis* (Poppe, 1880; Copepoda: Temoridae), *Acartia bifilosa* (Giesbrecht, 1881; Copepoda: Acartidae), *Bosmina coregoni* (Baird, 1957; Branchiopoda: Bosminidae), *Evadne nordmanni* (Lovén, 1836; Branchiopoda: Podonidae), and *Podon* sp. (Branchiopoda: Podonidae).

Perch and roach were collected by using Scientific Coastal Survey multimesh gillnets (Nippon Verkko Oy, Finland) with nine 5‐m‐long panels of different mesh size (mesh size ranging between 10 and 60 mm), which were deployed twice in late August in each bay. The nets were set around 16:00 and lifted between 07:00 and 08:00 the following morning. Nettings, methods of sacrifices, and design of all fish sampling strategies comply with the current laws of Sweden and were approved by the local ethics committee of the Swedish National Board for Laboratory Animals in Umeå (CFN, license no. A20‐14). For each bay, all caught fish from the two nettings were counted and measured (total length, to the nearest 1 mm and total weight, to the nearest 1 g), then pooled. The dorsal white muscle of selected individuals of perch and roach (total length ranged 17–22 cm) was dissected, freeze‐dried, and pulverized for analysis of FA and stable isotopes.

### 2.4 Fatty acid (FA) analysis

We used the methods described in Grieve and Lau (2018) and Lau et al. (2021) for FA analyses of zooplankton, benthic macroinvertebrates, and fish muscle samples. Briefly, FA from a homogenized freeze‐dried sample (1–10 mg) were extracted using 3:2 (v:v) hexane‐isopropanol solution. Deuterium‐labeled pentadecanoic acid (120 ng μL^−1^; C/D/N isotopes, Essex, UK) was used as the internal standard. Extracted FA were methylated by using 1:17:83 (v:v:v) trimethylsilyldiazomethane:isopropanol:dichloromethane. We quantified the concentrations of FA methyl esters in the samples by using a gas chromatography–mass spectrometry (7890A GC, Agilent Technologies; Pegasus® High Throughput TOF–MS, MI, United States) installed with a DB‐5 capillary column (length 30 m, internal diameter 250 μm, film thickness 0.25 μm; Agilent Technologies). A splitless injection of 1 μL was used for each sample. Individual FA were identified by using the Supelco 37 Component FAME Mix (Sigma‐Aldrich Sweden AB) and the Bacterial Acid Methyl Ester BAME Mix (Sigma‐Aldrich Sweden AB). Except EPA and DHA, we classified the sample FA into major groups: monounsaturated FA (MUFA), other PUFA (PUFA_other_, i.e., excluding EPA and DHA), short‐chain (ShortSAFA, with <20 carbon atoms) and long‐chain saturated FA (LongSAFA, with ≥20 carbon atoms). The PUFA_other_ were further separated into PUFA_ω3other_ and PUFA_ω6_ in the principal component analysis (see below). In total, we identified 31 FA and their contents in the samples were reported as mg FA g^−1^ dry weight. As bacterial FA constituted very small proportions (<0.1%) of the total FA content, they were excluded in the data analyses and finally, 27 FA were included in the analyses.

### 2.5 Stable isotope analysis

Approximately 1 mg of homogenized freeze‐dried benthic macroinvertebrates, fish dorsal white muscle, and zooplankton were weighed into tin capsules (Säntis Analytical) and analyzed for stable carbon and nitrogen isotopes (δ^13^C and δ^15^N) at the University of California Davis Stable Isotope Facility, USA. Samples were analyzed by using an elemental analyzer (PDZ Europa ANCA‐GSL) interfaced with an isotope ratio mass spectrometer (PDZ Europa 20‐20; Sercon Ltd.). The isotopic composition of the samples as denoted by δ^13^C and δ^15^N (in ‰) were calculated by using the equation: (*R*_sample_/*R*_standard_) − 1, where *R* = ^13^C/^12^C or ^15^N/^14^N. Ratios are relative to the international standards of Vienna PeeDee Belemnite for δ^13^C and air for δ^15^N. The long‐term standard deviations for δ^13^C and δ^15^N are 0.2‰ and 0.3‰, respectively.

### 2.6 Statistical analyses

All statistical analyses were performed by using R version 4.0.2 (R Development Core Team, 2017) with the package *vegan* version 2.5.7 (Oksanen et al., 2020) for all multivariate analyses. Data of the environmental variables (except pH and salinity) and the FA contents of all biological samples were log_10_‐transformed to meet the assumptions of normality and homoscedasticity. We used principal component analysis (PCA) to analyze the spatial and temporal variation in the water physiochemical characteristics of the bays. Since temporal FA data were not available for all taxa throughout the season (i.e., fish), we used PCA to examine only the spatial and taxonomic differences in the animal groups (i.e., zooplankton, benthic macroinvertebrates, and fish), and tested these differences with permutational multivariate analysis of variance (PERMANOVA; R function *adonis*, package *vegan*) using a Euclidean distance matrix. Prior to PERMANOVA, multivariate homogeneity of the FA data was confirmed (R function *betadisper*, package *vegan*, *p* > .05). Two‐way ANOVA and post hoc Tukey's HSD test were used to compare the spatial and temporal differences in DHA, EPA, and PUFA_other_ within individual prey taxa (Tables S1–S3). PUFA_other_ was dominated by the 18‐carbon PUFA, which are precursors for potential synthesis of EPA and DHA in consumers. We used *t*‐test to compare the DHA, EPA, and PUFA_other_ content between both perch and roach.

The invertebrates from benthic habitats and zooplankton from pelagic habitats generally differ in their FA composition and LC‐PUFA content (Lau et al., 2012, 2014). Roach and perch are able to consume both benthic and pelagic prey, and their trophic dependence on benthic versus pelagic prey potentially affects their LC‐PUFA acquisition. Initial statistical analysis showed that, for each bay, the δ^13^C of individual prey taxa did not differ significantly among sampling months (Table S4), thus samples of the same taxon from all months were used as replicates. We then used the δ^13^C data and a two‐end‐member mixing model to determine the relative pelagic and benthic reliance of our fish samples (Post, 2002; Vander Zanden & Vadeboncoeur, 2002). We used *Bosmina coregoni* and Gastropoda as the pelagic and benthic end members, respectively, in our model, as they had the lowest and highest δ^13^C, respectively, (i.e., covering the full δ^13^C range of all study animals) in all study bays (Figure S1a). *Bosmina coregoni* and Gastropoda also had the lowest δ^15^N among zooplankton taxa and benthic macroinvertebrates, respectively, at all sites (Figure S1b). Gastropods are commonly used as littoral or benthic end members for isotopic models in aquatic food web studies (Devlin, 2011; Vander Zanden et al., 2011). Among‐site and between‐species differences in benthic reliance of the fish were assessed by Kruskal–Wallis test followed by Bonferroni‐adjusted Dunn's pairwise comparisons, as the data were not normally distributed.

The FA retention factors were used as proxies to indicate the amount of specific FA (i.e., DHA, EPA, and PUFA_other_) accumulated in the fish relative to that in their dietary resources and were obtained by calculating the ratio between the FA content in fish and that in their potential diet (Hessen & Leu, 2006; Kainz et al., 2006). For example, the DHA retention factor is calculated as:

$$\mathrm{DHA}\;\text{retention factor}=\frac{{\mathrm{DHA}}_{\text{fish}}}{{\mathrm{DHA}}_{\text{diet}}}$$

where DHA_fish_ is the measured DHA content in fish. DHA_diet_ is the calculated overall dietary DHA content received by the fish from their benthic and pelagic prey. It was calculated by summing the DHA contents of both prey groups (benthic macroinvertebrates and zooplankton) that were weighted by the groups' relative contributions to the fish biomass (i.e., Reliance_benthic_ and Reliance_pelagic_; Scharnweber et al., 2021):

$${\mathrm{DHA}}_{\text{diet}}={\mathrm{Reliance}}_{\text{pelagic}}\times {\mathrm{DHA}}_{\text{zooplankton}}+{\mathrm{Reliance}}_{\text{benthic}}\times {\mathrm{DHA}}_{\text{benthos}}$$

where both Reliance_pelagic_ and Reliance_benthic_ were obtained by the isotopic mixing model based on δ^13^C of *Bosmina coregoni* and Gastropods at each site to indicate the relative amounts (i.e., proportions) of the pelagic and benthic prey groups, respectively, that had been assimilated into the fish biomass. DHA_zooplankton_ and DHA_benthos_ are the average DHA content among taxa of zooplankton and benthic macroinvertebrates (i.e., *L. balthica* and gastropods), respectively. The polychaete *Marenzellaria* sp. was excluded as a potential benthic prey source, as it lives in deeper sediment layers and is considered difficult to be accessed by perch and roach (Winkler, 1996). We also excluded chironomid larvae, as they are not preferred benthic prey by adult perch (Wagner et al., 2012) and roach in the northern Baltic Sea (Hansson, 1984; Lappalainen et al., 2001). When a given FA content in fish is similar to that of the diet, the FA retention factor is ≈1. A retention factor of >1 or <1 indicates the given FA content in fish is more concentrated or diluted, respectively, than the diet. We quantified the LC‐PUFA and PUFA_other_ availability (mg m^−2^) of prey resources at each site for spatial comparison. However, we did not have the zooplankton biomass data from our study sites and therefore these calculations were only limited to benthic macroinvertebrates.

Spatial differences in the FA retention factor of each fish species were tested with Kruskal–Wallis test followed by Bonferroni‐adjusted Dunn's pairwise comparisons, as the data were not normally distributed. We used redundancy analysis (RDA; R function *rda*, package *vegan*) based on the Euclidean distance matrix to examine the effects of taxon identity and site on FA retention in fish. The DHA, EPA, PUFA_other_, and SAFA (i.e., ShortSAFA + LongSAFA) retention factors were used as dependent variables in the RDA. We also included the SAFA retention factor in the RDA, as SAFA are commonly used as an energy reserve in animals. The benthic reliance (i.e., Reliance_benthic_) of fish was additionally included as a dependent variable to examine whether it varied between fish species and site, and whether it was associated with the FA retention factors. Followed by the RDA, we partitioned the variance (R function *varpart*, package *vegan*) explained by taxon identity and site to elucidate the main determinant of FA retention in fish.

## 3 RESULTS

### 3.1 Spatial and temporal variation of the water physiochemical characteristics

**FIGURE 2:**
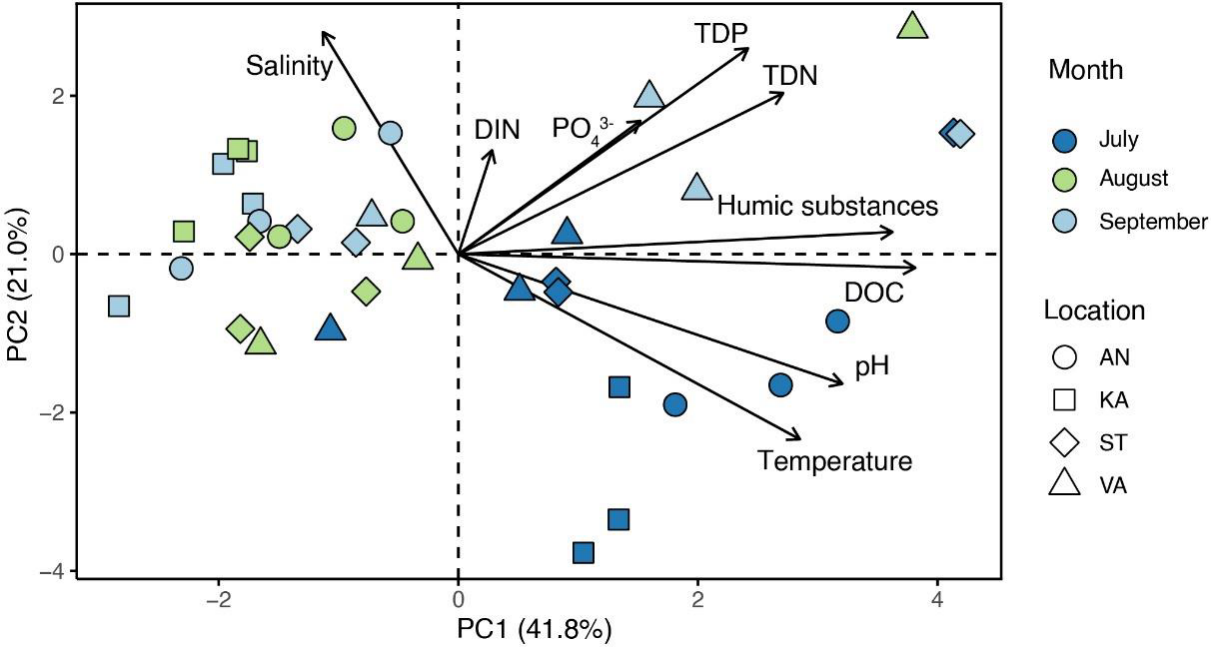
Principal component analysis (PCA) of water physicochemical characteristics in the study bays in July–September 2018. Variance percentages explained by the PCA axes are indicated in parentheses. AN, Ängerån; KA, Kalvarsskatan; ST, Stadsviken; and VA, Valviken. TDP, total dissolved phosphorus; TDN, total dissolved nitrogen; DOC, dissolved organic carbon; DIN, dissolved inorganic nitrogen; PO_4_^3‐^, phosphate. Except pH and salinity, the data were log_10_‐transformed before PCA.

Results of PCA indicated spatiotemporal variation in the water physiochemical characteristics of the bays, and the temporal variation was stronger than the spatial variation (Figure 2). The first axis (PC1) explained 41.8% of the total variance and was positively correlated with DOC, humic substances, pH, temperature, and TDN. The second axis (PC2) explained 21.0% of the total variance and was positively correlated with salinity and DIN. Samples from July were associated with higher temperature and pH and were clearly separated from the August and September samples, while the August and September samples overlapped on the PCA biplot. Yet, within‐site variation was particularly large at Valviken in August and at Stadsviken in both July and September.

### 3.2 Variation of major fatty acid groups in different taxa

**FIGURE 3:**
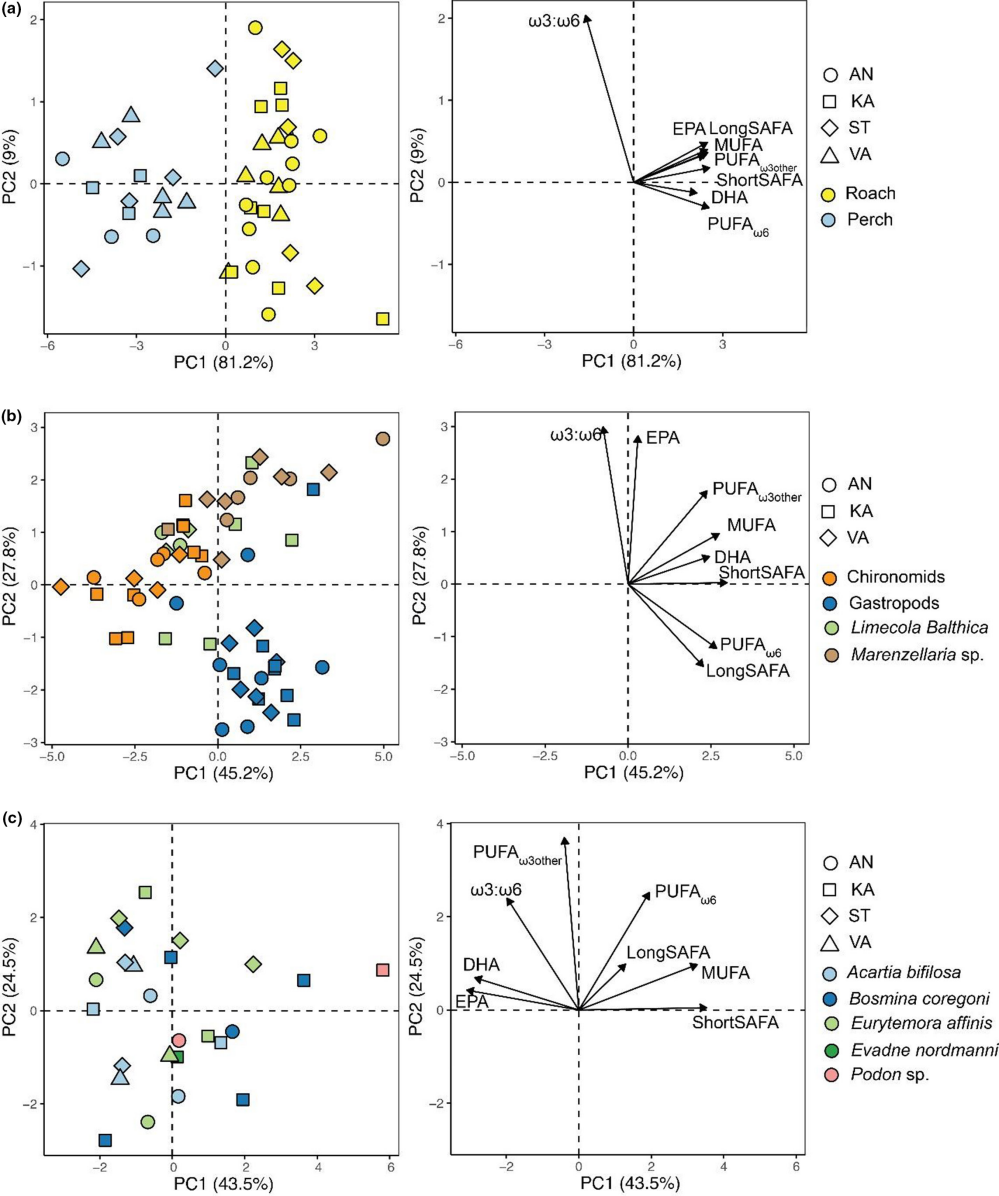
Principal component analysis (PCA) of FA contents (mg FA g^−1^ dry weight) in (a) fish, (b) benthic macroinvertebrates, and (c) zooplankton. Variance percentages explained by the PCA axes are indicated in parentheses. AN, Ängerån; KA, Kalvarsskatan; ST, Stadsviken and VA, Valviken. MUFA, monounsaturated FA; ShortSAFA, short‐chain saturated FA; LongSAFA, long‐chain saturated FA; PUFA_ω3other_, ω3 PUFA excluding DHA and EPA; PUFA_ω6_, ω6 PUFA. ω3:ω6, the ratio between ω3 and ω6 PUFA. All data were log_10_‐transformed before PCA.

Roach had higher content of all FA than did perch at all sites based on the PCA (Figure 3a), where PC1 explained a majority (81.2%) of the total variance and was positively correlated with most FA. In benthic macroinvertebrates, PC1 and PC2 explained 45.2% and 27.8% of the total variance, respectively (Figure 3b). Compared with other benthic macroinvertebrate taxa, both *L. balthica* and *Marenzellaria* sp. were rich in DHA, EPA, and PUFA_ω3other_, while the gastropods were rich in PUFA_ω6_ and LongSAFA but had low EPA. In zooplankton, PC1 and PC2 explained 43.5% and 24.5% of the total variance of the data, respectively (Figure 3c). Calanoid copepods (*E. affinis* and *A*. *bifilosa*) were generally rich in DHA and EPA. Among all animal taxa, there were significant taxonomic differences in their FA composition (PERMANOVA, *F*_11_,_111_ = 26.63, *p* < .001; Figure S2), while the spatial differences were not significant (Table S5). Both roach and perch had higher contents of EPA, DHA, and PUFA_ω3other_ than the benthic macroinvertebrates and zooplankton (Figure S2).

### 3.3 Benthic reliance of fish

**FIGURE 4:**
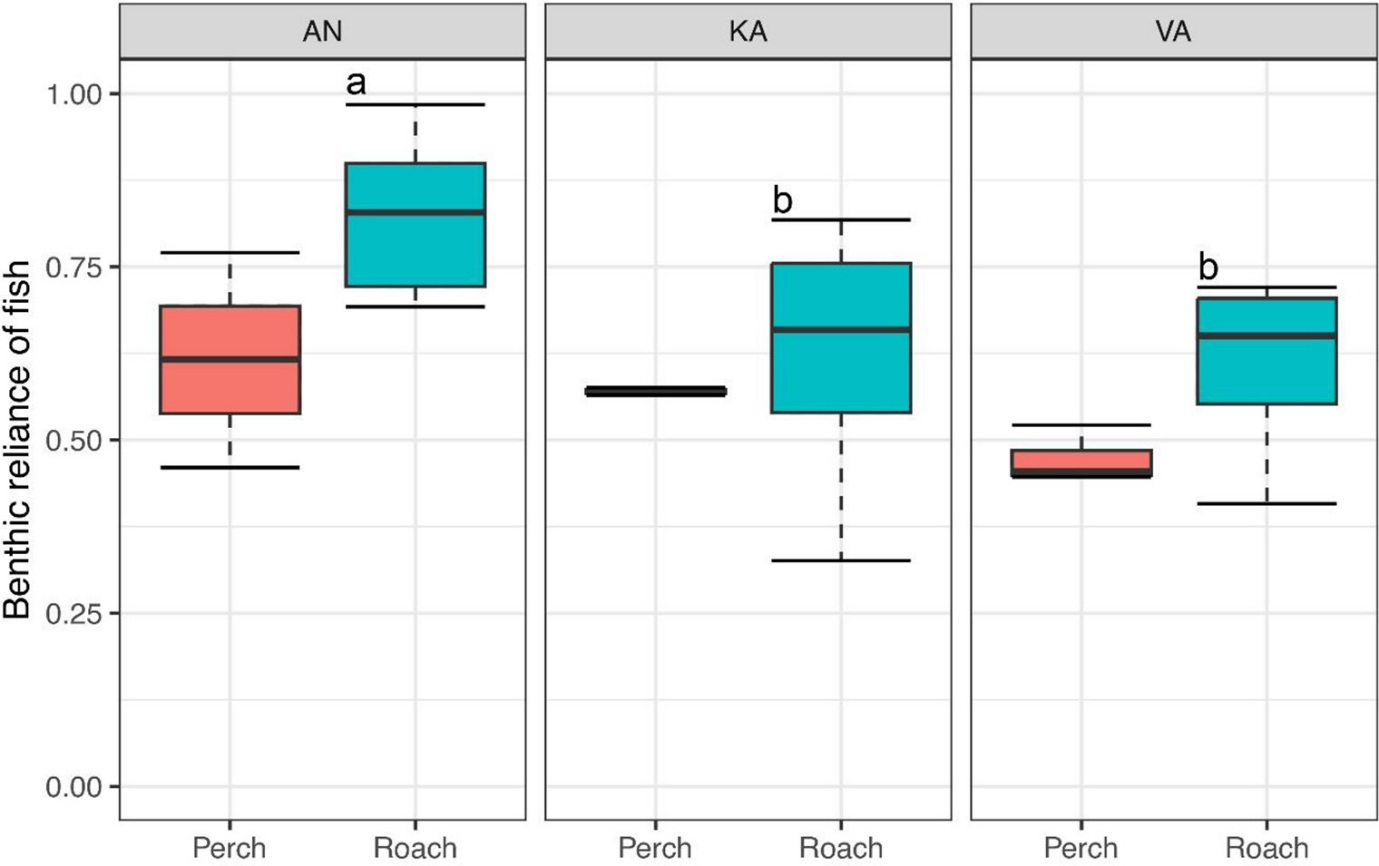
Trophic reliance on benthic prey of roach and perch at the study sites. A value >0.5 indicates greater reliance on benthic prey than pelagic prey. Line represents the median; box represents the upper (75%) and lower (25%) quantiles, and the whiskers represent maximum and minimum values. Different letters denote significant among‐site differences in benthic reliance of roach by Kruskal–Wallis test and Bonferroni‐adjusted Dunn's pairwise comparisons (*p* < .05). AN, Ängerån; KA, Kalvarsskatan and VA, Valviken.

Both roach and perch at Ängerån and Kalvarsskatan were more dependent on benthic prey (>50%) than pelagic prey (Figure 4). However, the benthic reliance of roach at Ängerån was significantly higher than at Kalvarsskatan and Valviken, while perch had similar benthic reliance across sites (Figure 4). Roach had significantly higher benthic reliance than perch (Kruskal–Wallis test, *p* < .05).

### 3.4 DHA, EPA and PUFA_other_ retention of fish

**FIGURE 5:**
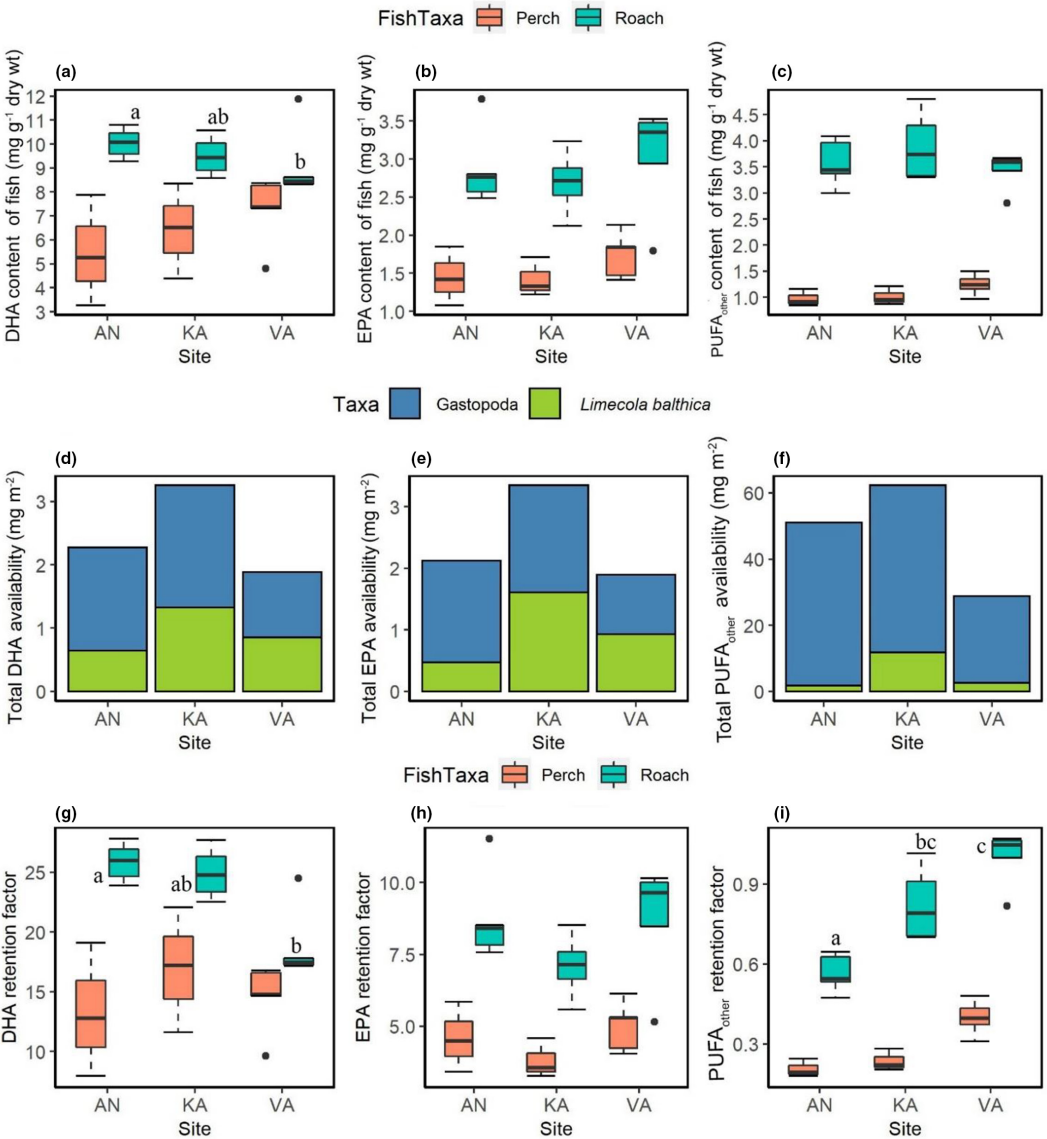
Upper panels: (a) DHA, (b) EPA, and (c) PUFA_other_ contents (mg g^−1^ dry weight) of fish. Middle panels: total availability (mg m^−2^) of (d) DHA, (e) EPA, and (f) PUFA_other_ in the major benthic prey resources (i.e., gastropods and *L. balthica*) for fish. Lower panels: retention factors of (g) DHA, (h) EPA, and (i) PUFA_other_ in fish, which are the ratios of these FA between fish and their prey from pelagic (i.e., zooplankton) and benthic habitats (i.e., *L. balthica* and Gastropods). Boxes with different letters indicate significant differences (*p* < .05) among sites for individual fish species by one‐way ANOVA followed by Tukey HSD (a–c) or Kruskal–Wallis test followed by Bonferroni‐adjusted Dunn's pairwise comparisons (g–i). AN, Ängerån; KA, Kalvarsskatan and VA, Valviken.

The DHA, EPA, and PUFA_other_ contents differed between roach and perch. Roach had significantly higher DHA, EPA, and PUFA_other_ contents than did perch in all locations (*t*‐tests: *p* < .05; Figure 5a–c). In both roach and perch, no spatial differences in their EPA and PUFA_other_ contents were found (Figure 5b,c). However, the DHA content of roach at Valviken was significantly lower than at Ängerån, while DHA content of roach at Kalvarsskatan was not different from that at these two sites (Figure 5a). Similarly, the total DHA, EPA, and PUFA_other_ availability in *L. balthica* and gastropods were lower at Valviken than at Ängerån and Kalvarsskatan (Figure 5d–f).

DHA and EPA retention factors of both roach and perch were much higher than their PUFA_other_ retention factors, suggesting that DHA and EPA were preferentially accumulated by the fish (Figure 5g–i). There was no significant spatial difference in EPA retention in both roach and perch (Figure 5h). However, the DHA and PUFA_other_ retention factors of roach were lower and higher, respectively, at Valviken than at Ängerån (Figure 5g,i).

**FIGURE 6:**
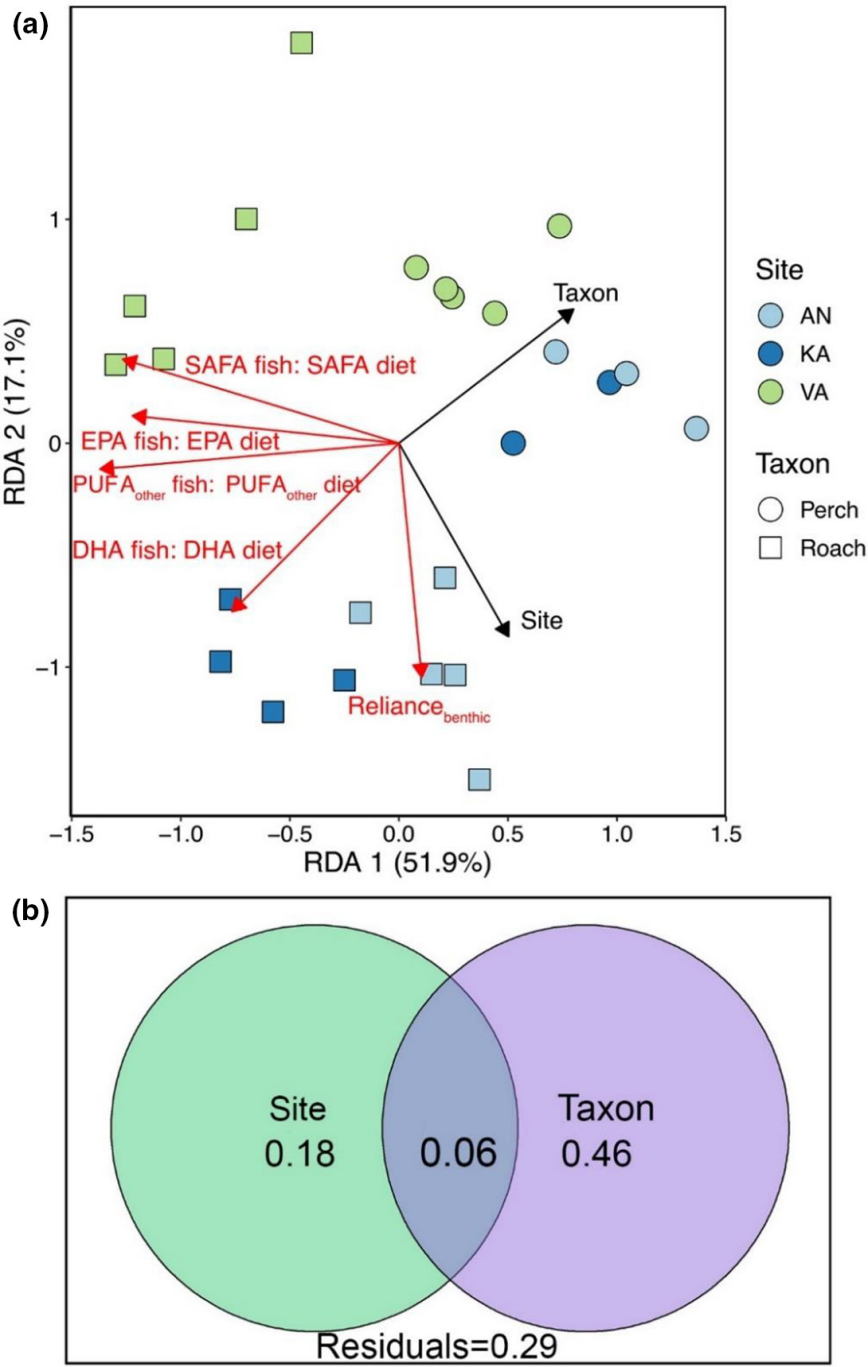
(a) Redundancy analysis (RDA; *p* < .01) of FA retention and benthic reliance of fish (red arrows) with the explanatory factors, that is, site and taxon identity (black arrows). Variance percentages explained by the RDA axes are indicated in parentheses. AN, Ängerån; KA; Kalvarsskatan and VA, Valviken. (b) Partial RDA showing the contribution of fish taxon identity and site to the total variance in fish FA retention and benthic reliance.

The RDA on FA retention factors of fish and underlying explanatory factors, that is, site and taxon identity, showed that the first axis explained 51.9% of the total variance and was mainly attributed to taxon identity, with higher DHA, EPA, PUFA_other_, and SAFA retention in roach than in perch (Figure 6a). The second axis explained 17.1% of the total variance and was mainly attributed to site effects, with generally lower benthic reliance of fish at Valviken than at Ängerån and Kalvarsskatan (Figure 6a). Fish DHA retention was positively correlated with their benthic reliance (Figure 6a). Taxon identity and site explained 46% and 18%, respectively, of the total variance in FA retention and benthic reliance of fish (Figure 6b), and their joint effect accounted for a relatively low proportion (6%) of the total variance.

## 4 DISCUSSION

The DHA and EPA retention was higher than the PUFA_other_ retention in both roach and perch, supporting our first prediction that these predators would retain the more physiologically important LC‐PUFA. The low retention of PUFA_other_ (i.e., precursor FA) in fish possibly reflects their limited biosynthesis of DHA and EPA. The intraspecific variation in DHA content of roach was likely related to the spatial differences in the nutritional quality of their diet. In particular, our results showed that roach had a greater demand for DHA than did perch, and the lower DHA availability in benthic prey at Valviken was coupled with lower benthic reliance and DHA content, resulting in lower DHA retention in roach at Valviken than at the other sites. The PUFA_other_ retention in roach was concurrently higher at Valviken, suggesting their stronger need for these precursors FA for DHA synthesis when dietary DHA availability was low. We did not find spatial differences in LC‐PUFA content and retention in perch, which could be attributed to their lower requirements for LC‐PUFA or their ability to maintain LC‐PUFA content despite differences in dietary LC‐PUFA supply. These results support our second and third hypotheses that LC‐PUFA requirements and retention of fish are taxon‐specific, and that habitat use and the spatial differences in nutritional quality and availability of prey contribute to intraspecific variation in LC‐PUFA retention of fish, especially for species that have high LC‐PUFA requirements.

### 4.1 Changes between DHA, EPA and PUFA_other_ retention of fish

The physiological need for DHA and EPA in fish depends on the fish traits that affect ecosystem functioning (e.g., habitat and prey selectivity (Scharnweber et al., 2016) and trophic position [Kainz et al., 2017]), and the traits that result from fish responses to environmental conditions (e.g., ontogeny and internal metabolic process [Chaguaceda et al., 2020; Závorka et al., 2022]), all of these traits likely differ among taxa. Previous studies have also indicated that dietary availability in DHA and EPA is an important determinant of their retention in consumers (Gladyshev et al., 2022; Napolitano, 1999). Both roach and perch in our study were generally more dependent on benthic (>50%) than pelagic prey, although benthic invertebrates are often considered as having a lower food quality compared to zooplankton (Gladyshev et al., 2018; Scharnweber et al., 2021; Vesterinen et al., 2021). The nutritional quality of benthic invertebrates, measured in terms of DHA and EPA content in our study was low compared to that in subarctic lakes (Vesterinen et al., 2021). Therefore, the observed high DHA and EPA retention in both fish species reflected the large mismatch between the low nutritional quality of their benthic prey and the high physiological demand for DHA and EPA in fish.

Our results showed that the DHA, EPA, and PUFA_other_ retention in fish is taxon‐specific. A recent meta‐analysis similarly showed that phylogeny largely determines the DHA and EPA content in fish (Gladyshev et al., 2018). The lower retention of DHA, EPA, and PUFA_other_ in perch than in roach indicates that the contents of these FA in perch were more similar to those in their major diet (i.e., benthic prey) than were roach, and the benthic prey quality was therefore higher for perch than for roach. Yet the benthic reliance was generally higher in roach than in perch, suggesting that roach were particularly specialized on the benthic prey resources despite their relatively low nutritional quality, while perch were more flexible in diet in northern aquatic ecosystems. Thus, environmental changes that affect the benthic prey quality and quantity may have larger impacts on the nutrition and health of roach than perch in these ecosystems.

Irrespective of taxa, we also found that DHA was more retained than EPA in fish. DHA retention in both perch and roach can be related to their habitat use and site‐specific characteristics (e.g., the nutritional quality of diet at each site), as these variables were correlated with DHA retention in the RDA plot (Figure 6a). Fish generally need more DHA than EPA, as DHA is more important in fish metabolism and reproduction (Parrish, 2009; Pilecky et al., 2021, 2022). A lower retention of EPA than DHA in fish could potentially result from elongation of EPA to DHA and/or a higher dietary supply of EPA than DHA (Guo et al., 2017). Although we did not find direct evidence for elongation of EPA in fish, the average EPA:DHA ratio in benthic macroinvertebrates (i.e., the major diet of fish) in our study was >1, indicating that the benthic macroinvertebrates had a higher content of EPA than DHA. Also, a modeling study showed that bioconversion of EPA to DHA is the major pathway for yellow perch (*Perca flavescens* [Mitchill, 1814]) to obtain DHA, which caused its lower EPA retention than DHA retention (Sawyer et al., 2016). Therefore, the bioconversion of dietary EPA to DHA possibly had contributed to the lower EPA retention than DHA retention in the fish in our study. Yet, PUFA_other_ retention factors of both roach and perch in our study were <1 and much lower than their EPA and DHA retention, indicating that PUFA_other_ were not efficiently retained in the fish. Both fish species likely have limited requirements for PUFA_other_ for DHA and EPA synthesis (Twining et al., 2019).

### 4.2 Spatial variation in DHA, EPA and PUFA_other_ retention of fish

Roach had significantly lower DHA retention at Valviken than at the other sites. This lower DHA retention in roach was probably linked to the lower dietary DHA availability in benthic macroinvertebrates at Valviken, and the lower benthic reliance of roach at this site than at the other sites. These results imply that reduction in nutritional quality of prey could alter the predator–prey relationships, especially for predators that have more specialized prey and/or habitat use, ultimately affecting their role in food‐web and ecosystem functioning (Závorka et al., 2022). Recent studies indicated that increases in water temperature caused by climate change will reduce DHA and EPA production by primary producers and their transfer to primary consumers in aquatic ecosystems worldwide (Hixson & Arts, 2016; Holm et al., 2022). The subarctic marine ecosystems such as the northern Baltic Sea are increasingly threatened by climate change (Andersson et al., 2015). With ongoing climate change, the nutritional quality especially the production and availability of DHA and EPA at the lower trophic levels may further be diminished (Bandara et al., 2022), consequently causing repercussions for the prey quality and health of the fish predators.

The benthic macroinvertebrates at Valviken had lower DHA, EPA, and PUFA_other_ availability than those at Ängerån in our study. However, contents of these FA in perch did not follow the same spatial patterns as in the benthic macroinvertebrates and did not differ among sites. These results suggest that the low DHA, EPA, and PUFA_other_ availability at Valviken could have already met the perch requirements for these FA or that perch obtained these FA mainly from other prey resources. Perch are known to shift diet when they increase in size, with a higher degree of piscivory in larger individuals (Vrede et al., 2011). Based on stable isotopes and FA, Scharnweber and Gårdmark (2020) found that perch can be strongly dependent on prey fish resources in coastal Baltic Sea ecosystems much further south of our study sites. Perch had higher δ^15^N than roach in all our study bays (Figure S1a,b), indicating the higher trophic position of perch than roach. Thus, we conjecture that perch also obtained LC‐PUFA from prey fish in our study. However, as prey fish likely have higher LC‐PUFA concentrations than zooplankton and benthic macroinvertebrates (Scharnweber & Gårdmark, 2020), the expected LC‐PUFA retention factors of perch from prey fish would be lower than that from zooplankton and benthic macroinvertebrates. Piscivory of perch thus will not affect our conclusion that roach had a higher LC‐PUFA demand and retention than perch but might have confounded the observed spatial patterns in LC‐PUFA retention of perch.

Perch in our study might also have obtained DHA, EPA, and PUFA_other_ from zooplankton, which generally have higher DHA and EPA content than do the benthic macroinvertebrates (Lau et al., 2012). However, the FA composition of fish was different from that of both benthic and pelagic prey in our study (Figure S2). We therefore do not exclude the possibility of limited biosynthesis of DHA and EPA from dietary precursor FA (Chaguaceda et al., 2020). It has been shown that perch fed low LC‐PUFA diet are able to convert precursor FA into EPA and DHA (Henrotte et al., 2011). To date, other than aquaculture studies, very few studies have focused on DHA and EPA biosynthesis in wild fish populations (Guo et al., 2017; Závorka et al., 2022). Compound‐specific stable isotope analysis can be useful for identifying the source of DHA and EPA (either from internal metabolism or from diet) in fish (Scharnweber et al., 2021). Controlled feeding experiments would also assist in differentiating the importance of internal metabolism and diet on DHA, EPA, and PUFA_other_ retention in fish.

## 5 CONCLUSION

The DHA and EPA retention in fish was taxon‐specific and was higher in roach than in perch. Spatial differences in DHA content and retention were found in roach only, which were likely attributed to the among‐site differences in their benthic reliance and the nutritional quality of prey. Yet, DHA was strongly retained in both fish species due to its physiological importance. Although DHA and EPA availability in the prey resources was limited, both fish species were able to accumulate DHA and EPA. Climate change together with other human‐caused environmental stressors will likely alter the algal assemblages and lower their LC‐PUFA supply for aquatic food webs (Bandara et al., 2022; Guo et al., 2017). We advocate further investigations on how environmental changes would affect the nutritional quality of the basal trophic level, and their subsequent impacts on LC‐PUFA retention, trophic ecology, and performance of individual fish species, especially those with high LC‐PUFA requirements.

## AUTHOR CONTRIBUTIONS

**Tharindu Bandara:** Formal analysis (lead); writing – original draft (lead); writing – review and editing (equal). **Sonia Brugel:** Conceptualization (lead); methodology (lead); writing – review and editing (equal). **Agneta Andersson:** Conceptualization (lead); funding acquisition (lead); methodology (lead); supervision (lead); writing – review and editing (equal). **Danny Chun Pong Lau:** Conceptualization (lead); methodology (lead); supervision (lead); writing – review and editing (equal).

## CONFLICT OF INTEREST STATEMENT

The authors have no conflict of interest to declare.

## Supporting information


Appendix S1.

## Data Availability

Data available from Mendeley data digital repository. https://data.mendeley.com/datasets/r7hcvhmxy3/draft?a=253bcdef‐22e9‐4ba9‐bbca‐060f47e441fa.
