# Supplementary material for: Retention of essential fatty acids in fish differs by species, habitat use and nutritional quality of prey
Source: Ecol Evol. 2023 Jun 1;13(6):e10158. doi: 10.1002/ece3.10158 (PMC10234757; doi:10.1002/ece3.10158)
Supplement: Supplementary file 1 — Appendix S1. [file ECE3-13-e10158-s001.docx]

1. **Supporting information**

Table S1. Summary of two-way ANOVA results on the spatial and temporal variation of DHA content (mg g^-1^ dry weight) in each benthic macroinvertebrate and zooplankton taxon.

|  |  | DHA | | | |
| --- | --- | --- | --- | --- | --- |
|  |  | Between sites | | Between months | |
|  |  | F | *P* | F | *P* |
| *Limecola balthica* | 9 | 0.34 | 0.72 | 1.33 | 0.28 |
| *Marenzellaria* sp. | 13 | 1.61 | 0.24 | 20.32 | **<0.05^*^ (Jul>Sep)** |
| Gastropoda | 21 | 0.05 | 0.95 | 0.18 | 0.67 |
| Chironomids | 17 | 0.47 | 0.64 | 13.66 | **<0.05* (Jul>Sep)** |
| *Bosmina coregoni* | 6 | 0.77 | 0.54 | 0.23 | 0.81 |
| *Acartia bifilosa* | 8 | 0.18 | 0.91 | 0.65 | 0.56 |
| *Eurytemora affinis* | 9 | 0.03 | 0.99 | 63.44 | **<0.05* (Aug=Sep>Jul)** |

Table S2. Summary of two-way ANOVA results on the spatial and temporal variation of EPA content (mg g^-1^ dry weight) in each benthic macroinvertebrate and zooplankton taxon.

|  |  | EPA | | | |
| --- | --- | --- | --- | --- | --- |
|  |  | Between sites | | Between months | |
|  | n | F | *P* | F | *P* |
| *Limecola balthica* | 9 | 0.03 | 0.97 | 7.32 | 0.03 |
| *Marenzellaria* sp. | 13 | 2.00 | 0.18 | 9.25 | 0.01 |
| Gastropoda | 21 | 0.018 | 0.98 | 0.67 | 0.42 |
| Chironomids | 17 | 0.73 | 0.5 | 0.14 | 0.71 |
| *Bosmina coregoni* | 6 | 0.62 | 0.59 | 0.03 | 0.97 |
| *Acartia bifilosa* | 8 | 0.17 | 0.91 | 1.94 | 0.24 |
| *Eurytemora affinis* | 9 | 0.07 | 0.97 | 71.78 | **<0.05* (Aug=Sep>Jul)** |

Table S3. Summary of two-way ANOVA results on the spatial and temporal variation of PUFA_other_ content (mg g^-1^ dry weight) in each benthic macroinvertebrate and zooplankton taxon.

|  |  | | PUFA_other_ | | | |
| --- | --- | --- | --- | --- | --- | --- |
|  |  | | Between sites | | Between months | |
|  | | n | F | *P* | F | *P* |
| *Limecola balthica* | | 9 | 9.64 | 0.01 | 0.14 | 0.71 |
| *Marenzellaria* sp. | | 13 | 2.63 | 0.12 | 3.96 | 0.07 |
| Gastropoda | | 21 | 0.35 | 0.70 | 0.04 | 0.83 |
| Chironomids | | 17 | 2.79 | 0.09 | 0.37 | 0.55 |
| *Bosmina coregoni* | | 6 | 0.37 | 0.71 | 4.19 | 0.13 |
| *Acartia bifilosa* | | 8 | 0.06 | 0.97 | 0.12 | 0.88 |
| *Eurytemora affinis* | | 9 | 1.34 | 0.36 | 3.28 | 0.10 |

Table S4: Summary of Kruskal Wallis test results on δ^13^C of each benthic macroinvertebrate and zooplankton taxon.

|  |  | Between months | |
| --- | --- | --- | --- |
|  | n | H | *P* |
| *Limecola balthica* | 8 | 0.55 | 0.45 |
| *Marenzellaria* sp. | 14 | 0.2 | 0.65 |
| Gastropoda | 16 | 0.04 | 0.82 |
| Chironomids | 17 | 0.009 | 0.92 |
| *Bosmina coregoni* | 4 | 2.7 | 0.25 |
| *Acartia bifilosa* | 8 | 5.57 | 0.1 |
| *Eurytemora affinis* | 7 | 0.50 | 0.5 |

Table S5: PERMANOVA results on the effects of taxon identity, site and their interaction (Taxon identity × Site) on the FA composition of zooplankton, benthic macroinvertebrates, and fish.

| Term | Df | SS | R^2^ | F | *P* |
| --- | --- | --- | --- | --- | --- |
| Taxon identity | 11 | 20785.2 | 0.67 | 26.63 | <0.001 |
| Site | 3 | 386.2 | 0.01 | 1.81 | 0.08 |
| Taxon identity × Site | 20 | 2019.7 | 0.06 | 1.42 | 0.09 |


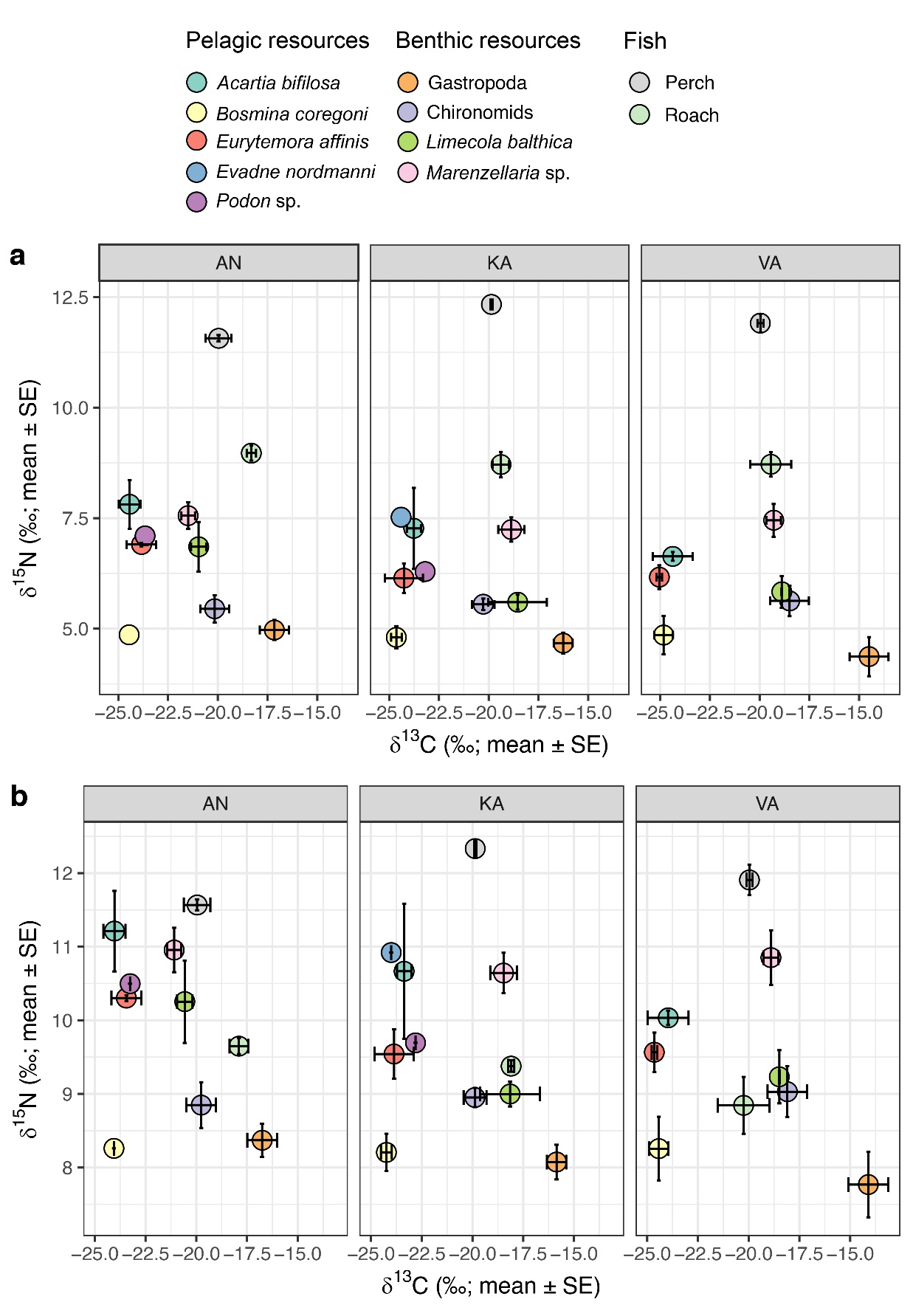


Figure S1: a) Stable isotope biplot (mean ± SE) of all study animal taxa from different sampling periods. Isotopic values of all prey resources do not include the trophic fractionations. b) Stable isotope biplot (mean ± SE) of all study animal taxa from different sampling periods including trophic fractionations for prey resources. Trophic fractionation and variability were obtained from (Post, 2002). AN, Ängerån; KA, Kalvarsskatan and VA, Valviken.

**
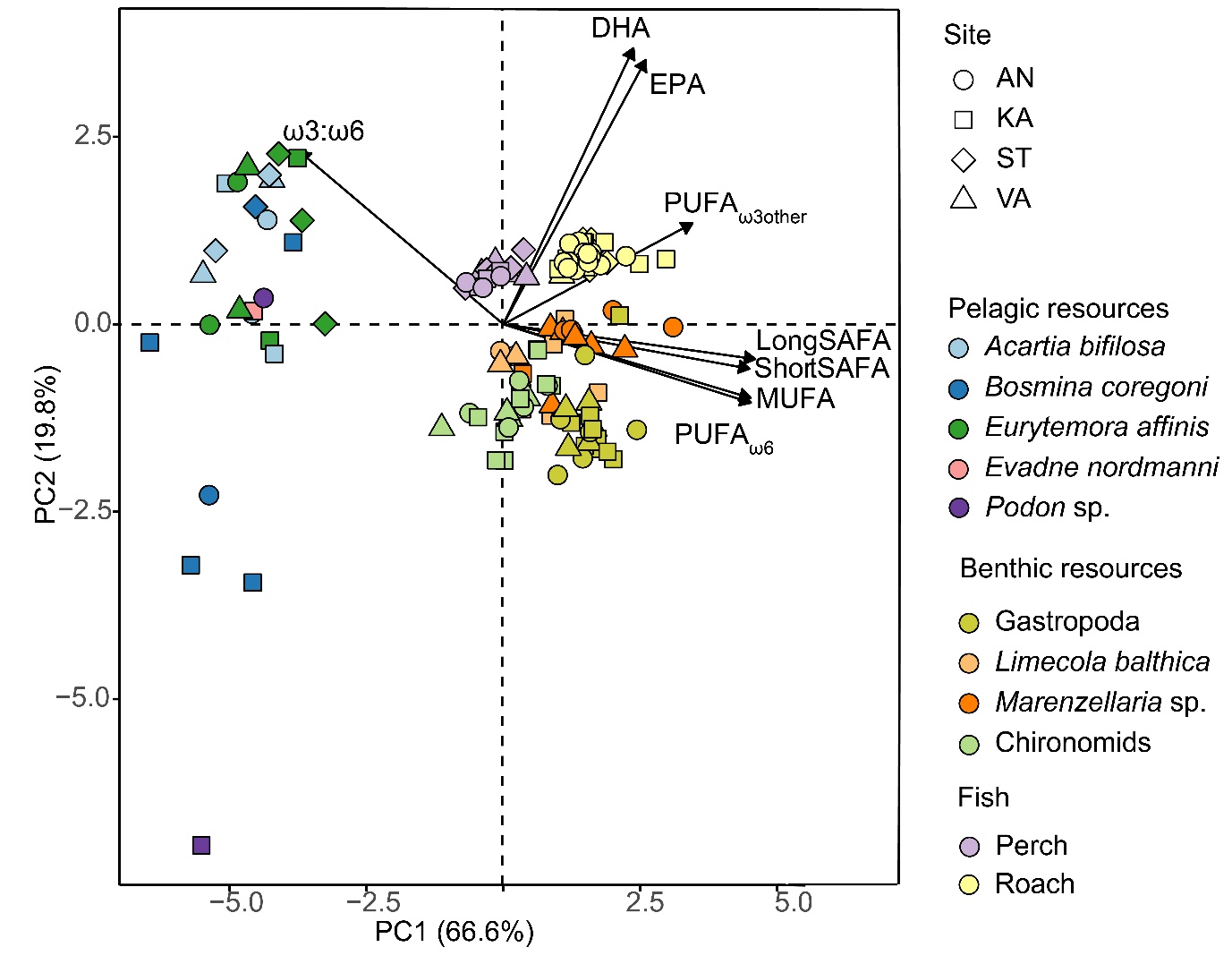
**

Fig. S2. Principal component analysis (PCA) of major FA groups (mg FA g^-1^ dry weight) in all taxa. Variance percentages explained by the PCA axes are indicated in parentheses. AN, Ängerån; KA, Kalvarsskatan; ST, Stadsviken; and VA, Valviken. MUFA, monounsaturated FA; ShortSAFA, short-chain saturated FA, LongSAFA, long-chain saturated FA; PUFA_ω3other_, ω3 PUFA excluding DHA and EPA; PUFA_ω6_, ω6 PUFA; ω3:ω6, the ratio between ω3 and ω6 PUFA. All data were log_10_-transformed before PCA.
